# Supplementary material for: Defective hierarchical porous copper-based metal-organic frameworks synthesised via facile acid etching strategy
Source: Sci Rep. 2019 Jul 26;9:10887. doi: 10.1038/s41598-019-47314-1 (PMC6659662; doi:10.1038/s41598-019-47314-1)
Supplement: Supplementary file 1 — Supplementary Information [file 41598_2019_47314_MOESM1_ESM.docx]

**Supplementary Information for**

**Defective hierarchical porous copper-based metal-organic frameworks synthesised via a facile acid etching strategy**

Huan V. Doan,^1,2^ Asel Sartbaeva,^3^ Jean-Charles Eloi,^4^ Sean Davis^5^ and Valeska P. Ting^*1^

^1^Department of Mechanical Engineering, University of Bristol, Bristol BS8 1TH, UK.

^2^Department of Oil Refining and Petrochemistry, Faculty of Oil and Gas, Hanoi University of Mining and Geology, Duc Thang, Bac Tu Liem, Hanoi, Vietnam.

^3^Department of Chemistry, University of Bath, Claverton Down, Bath, BA2 7AY, UK.

^4^Chemical Imaging Facility, School of Chemistry, University of Bristol, Bristol BS8 1TS, UK.

^5^School of Chemistry, University of Bristol, Bristol BS8 1TS, UK.

*e-mail: [v.ting@bristol.ac.uk](mailto:v.ting@bristol.ac.uk)

**Table of contents**

[1. Experimental 1](#_Toc536450760)

[2. Characterisation techniques 3](#_Toc536450761)

[3. Additional characterisation data and discussion 4](#_Toc536450762)

# **1. Experimental**

Table S1. Chemicals used.

| No. | Name and supplier | Purity | Formula |
| --- | --- | --- | --- |
| 1 | Methanol (Sigma-Aldrich) | 99.9% | CH_3_OH |
| 2 | Dimethyl sulfoxide (DMSO) (Sigma-Aldrich) | 99.9% | (CH_3_)_2_SO |
| 3 | Orthophosphoric acid (Honeywell) | 85-90% | H_3_PO_4_ |
| 4 | Basolite® C 300 (HKUST-1) (Sigma-Aldrich) | NA | C_18_H_6_Cu_3_O_12_ |
| 5 | Deuterium chloride (Sigma-Aldrich) | 99 atom% D | DCl |
| 6 | Dimethyl sulfoxide-d_6_ (Sigma-Aldrich) | 99.9 atom% D | (CD_3_)_2_SO |


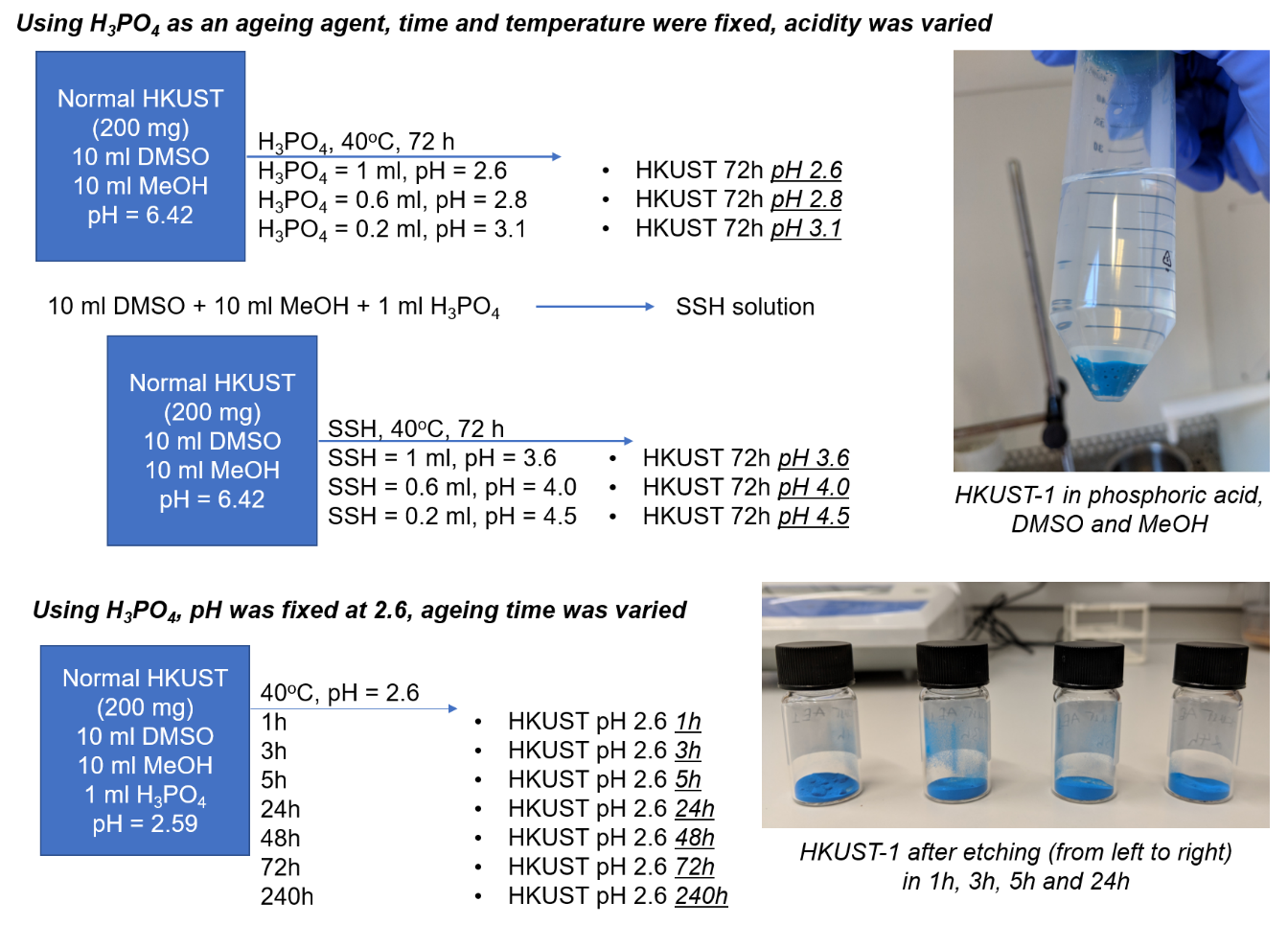


Figure S1. Experimental details of HKUST-1 etchied in phosphoric acid using DMSO and MeOH as dilute solvents.


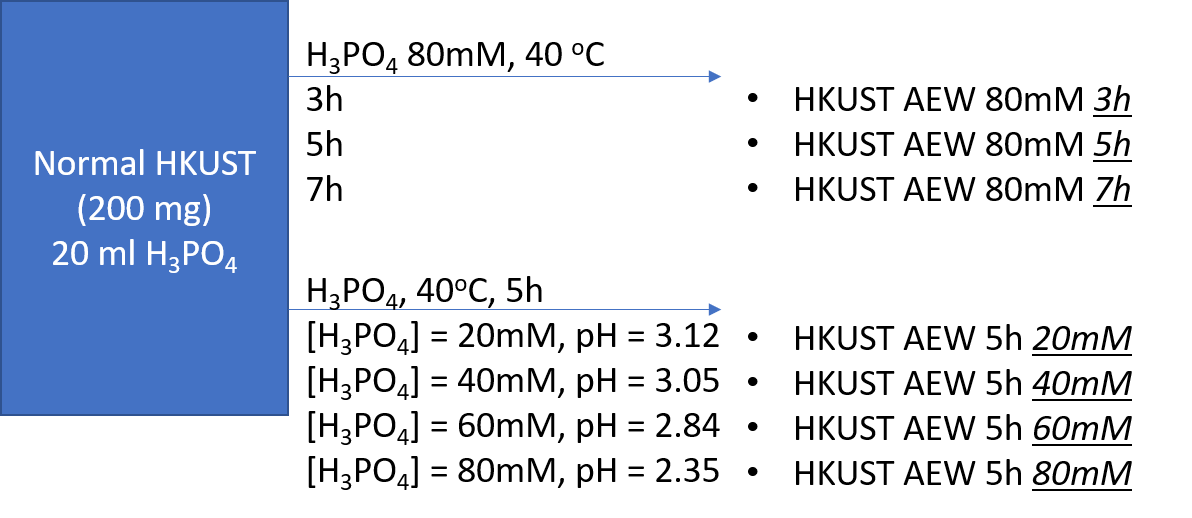


*Figure S2.* *Experimental details of HKUST-1 etched in phosphoric acid using water as a dilute solvent (HKUST AEW)*

At the highest concentration investigated in this study (200 mg HKUST etching in 20 ml phosphoric solution at pH 2.6), 0.15 mmol of H^+^ from the phosphoric acid was available to interact with 1 mmol of copper cations in HKUST-1. As the etching is due to the presence of H_3_PO_4_, increasing the solution volume with the same pH (i.e. the same concentration of H^+^, but increasing the number of moles of H^+^ available) could be expected to lead to a greater extent of etching (similar to the effect of leaving a sample in the SSH solution for longer).

# **2. Characterisation techniques**

Powder X-ray Diffraction analyses were performed on a BRUKER AXS D8-Advance instrument with Vantec-1 detector using Cu Kα (λ = 1.5418 Å) as the source of X-ray radiation, in flat plate geometry, spinner speed 15 rpm, at 21 ^o^C. The two theta range, between 2 and 60 degrees, was used with a 0.02 degree interval for collection and 20 minute scans.

Gas sorption isotherms were determined using nitrogen sorption at 77 K with a Micromeritics 3-Flex volumetric gas sorption analysis system. Samples were degassed at 120 °C under dynamic high vacuum (10^-6^ mbar) over 6 hours prior to analysis. The total pore volume was taken at the end of the filling of the pore. Surface area was determined by the BET method according to Bristish Standards, with relative pressure (P/P_o_) selected considering the Rouquerol consistency criterion between values of 0 and 0.3.

Scanning electron microscopy (SEM) analysis was perfomed on a JSM-IT300 (JEOL, Japan) at 5 kV with a 10.8 mm working distance and magnifications from 350 to 40,000 times. The samples were sputtered with a thin layer of silver (10 nm) and imaged with a secondary electron detector.

Thermogravimetric Analysis (TGA) were carried out in a thermogravimetric analyser (TGAQ500, TA-instruments) at the Material and Chemical Characterisation Facility, University of Bath. The temperature ramping was 5 °C min^−1^ from room temperature to 600 °C in 100 ml min^−1^ of air.

^31^P nuclear magnetic resonance (NMR) spectra were recorded at 298 K on a Bruker Advance 300 MHz Ultrashield NMR spectrometer at the Department of Chemistry, University of Bristol. ^1^H NMR spectra were referenced to the residual *protio* peaks at δ 2.50 ppm for DMSO-d6. The samples were digested in 0.1 ml deuterium chloride (DCl, 99 atom% D) and 3 ml anhydrous dimethyl sulfoxide-d_6_ (DMSO-d_6_, 99 atom% D) before carrying out the experiment.

# **3. Additional characterisation data and discussion**


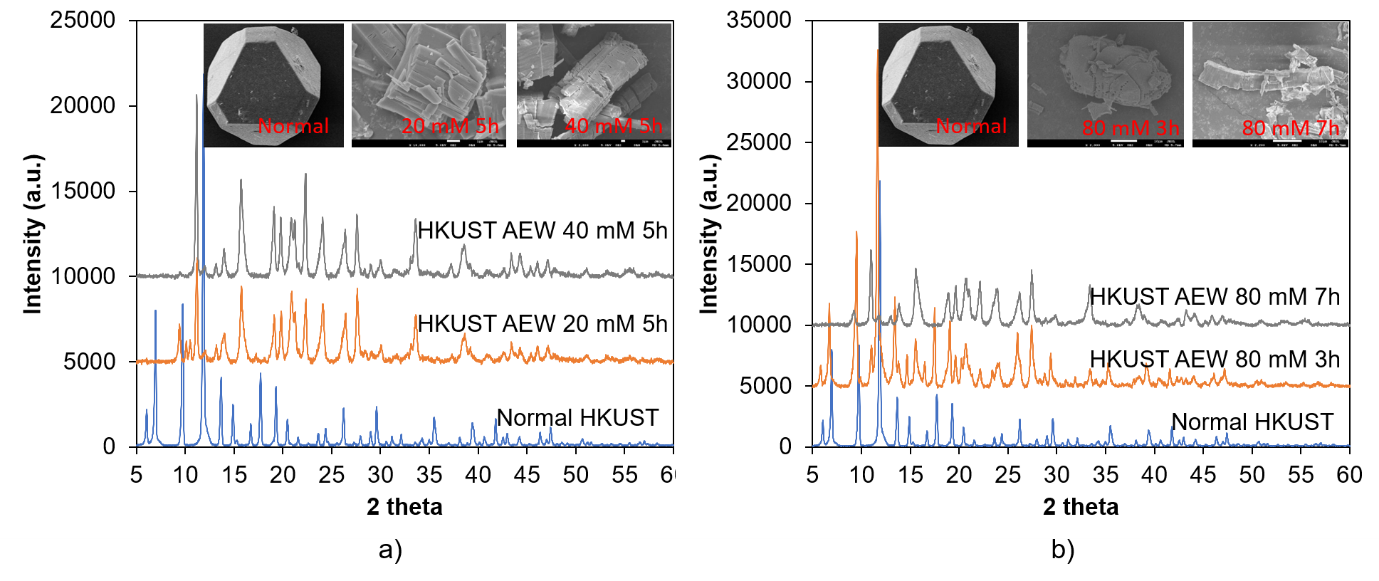


*Figure S3.* *PXRD and SEM results of HKUST-1 etched in phosphoric acid using water as a dilute solvent (HKUST AEW) in different concentrations (a) and times (b).*


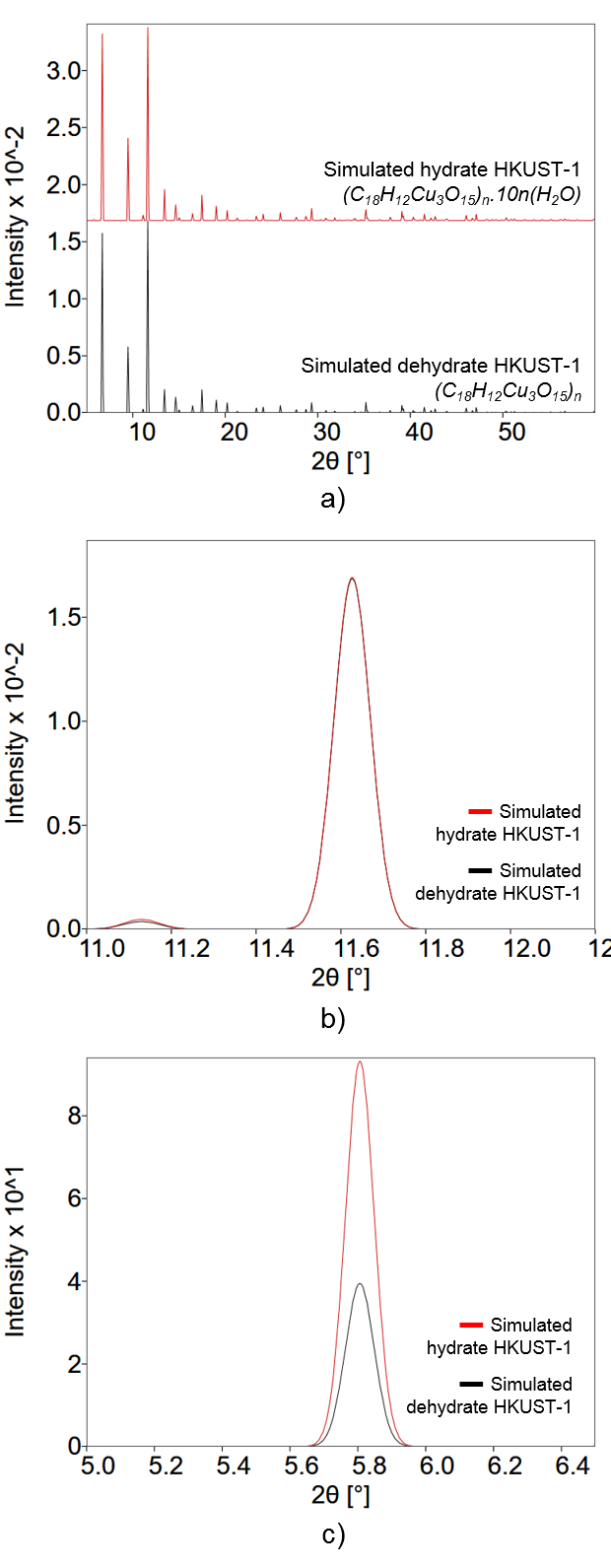


*Figure S4. PXRD results of simulated hydrate and dehydrate HKUST-1 generated by CrystalMaker and CrystalDiffract, normalised to the height of the peak at 11.6 degrees 2*$\theta$*. a) PXRD of both samples in 5-60 degrees 2*$\theta.$*b) Peaks at 11.6 degrees 2*$\theta$*in both patterns are identical. c) Peak at 5.8 degrees 2*$\theta$ *shown in hydrate HKUST-1 has much higher intensity than the dehydrated state.*

*Table S2. BET surface areas of HKUST-1 (measured via N_2_ sorption at 77 K) etching in phosphoric acid using water as a dilute solvent (HKUST AEW) at different concentrations.*

|  | Normal HKUST | HKUST AEW 5h 20mM | HKUST AEW 5h 40mM | HKUST AEW 5h 60mM | HKUST AEW 5h 80mM |
| --- | --- | --- | --- | --- | --- |
| BET surface area (m^2^ g^-1^) | 2208.04 ± 1.62 | 993.54 ± 0.65 | 17.64 ± 0.44 | 25.27 ± 0.89 | 17.06 ± 0.55 |

*Table S3. BET surface areas of* *HKUST-1 (measured via N_2_ sorption at 77 K) etching in phosphoric acid using water as a dilute solvent (HKUST AEW) in different times.*

|  | Normal HKUST | HKUST AEW 80mM 3h | HKUST AEW 80mM 5h | HKUST AEW 80mM 7h |
| --- | --- | --- | --- | --- |
| BET surface area (m^2^ g^-1^) | 2208.04 ± 1.62 | 33.44 ± 1.06 | 17.06 ± 0.55 | 33.47 ± 1.26 |

*Table S4. Gas sorption results of HKUST-1 etching in phosphoric acid using DMSO and MeOH as dilute solvents at different concentrations and times.*

|  | BET surface area (m^2^ g^-1^) | Total pore volume (cm^3^ g^-1^) |
| --- | --- | --- |
| *For different concentrations* | | |
| Normal HKUST | 2208 | 0.899 |
| HKUST 72 h pH 6.4 | 2088 | 0.913 |
| HKUST 72h pH 4.5 | 1743 | 0.737 |
| HKUST 72h pH 4.0 | 1730 | 0.715 |
| HKUST 72h pH 3.6 | 1538 | 0.701 |
| HKUST 72h pH 3.1 | 1525 | 0.625 |
| HKUST 72h pH 2.8 | 1523 | 0.623 |
| HKUST 72h pH 2.6 | 1455 | 0.585 |
| *Over different times* | | |
| HKUST pH 2.6 1h | 1523 | 0.665 |
| HKUST pH 2.6 3h | 1623 | 0.702 |
| HKUST pH 2.6 5h | 1616 | 0.689 |
| HKUST pH 2.6 48h | 1578 | 0.640 |
| HKUST pH 2.6 72h | 1455 | 0.585 |
| HKUST pH 2.6 240h | 1117 | 0.520 |

It was previously shown that phosphoric acid with molecular diameter of 0.61 nm^1^ is a very promising diffusing agent in some water stable MOFs for example MIL-100(Fe)^2^ to create a hollowing out structure via selective acid etching. HKUST-1 MOF, which has larger primary pores (d ~ 0.83 - 1.26 nm) and smaller secondary pore (d ~ 0.51 nm) (Figure S5) could be a very promising candidate to employ this process, regrettably, this MOF degrades in water under standard aqueous acid etching conditions. In this research, a mixture of DMSO and MeOH was systematically employed to control the concentration of H_3_PO_4_, enabling the mechanism of selective acid etching to be applied to MOFs with limited water stability.


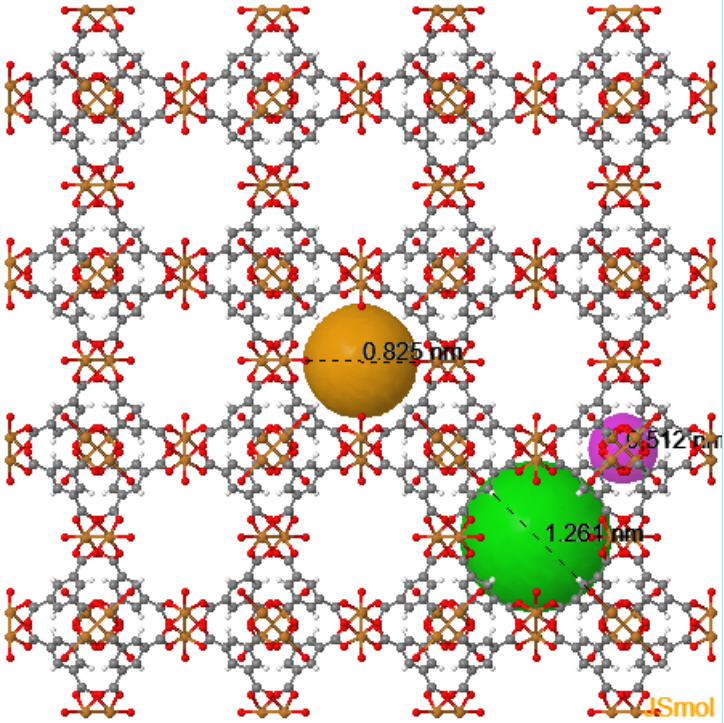


*Figure S5. Structure of HKUST-1 visualised using ChemTube3D.*^3^ *The spheres represent the pore sizes within the framework.* *The smaller secondary pore is represented by the purple sphere (0.512 nm, measured from two C atoms in the benzene rings) and the larger primary pores can be represented by either the orange sphere (0.825 nm*, *measured as the distance between the O atoms in the two opposing Cu paddlewheels) or the green sphere (1.261 nm, measured between the carbons of the two benzene rings)*

In this study, thermogravimetric analysis (TGA) was used to determine linkers or clusters missing in defect formation. This method was employed previously to study defect-engineered MOFs such as HKUST-1^4–7^, UiO-66^8^, NU-125^5^ and PCN-125^9^. For example, Taddei *et al.* observed that defected UiO-66 had smaller weight loss between 300 and 450 ^o^C, indicating the existence of missing-linker defects in the framework.^8^ Inspired by this research, TGA spectra of all HKUST-1 samples were collected and normalised to the weight loss at 600 ^o^C, allowing us to see the differences of linkers which might have been removed in the region between 250 and 300 ^o^C. From Figure S6, it can be seen that most etched samples possess missing-linker defects demonstrated by less weight loss than the normal HKUST-1, however, there is no obvious evidence to show that the number of linkers and clusters missing in HKUST acid etching depends on time or acidity. In addition, there was no peak for phosphorous compounds appearing in ^31^P NMR of samples etched in phosphoric acid using DMSO and MeOH as dilute solvents (Figure S7), indicating that there were no linker vacancies or partial linker decoordination by PO_4_^3-^. The phosphate anions were removed after washing out the sample 3 times with MeOH.

Figure S6. TGA results of HKUST-1 etching in phosphoric acid using DMSO and MeOH as dilute solvents at different concentrations (a) and times (b). Increasing time and decreasing pH are illustrated by the colours from lightest to darkest.

*Figure S7. ^31^P NMR of HKUST-1 etching in phosphoric acid using DMSO and MeOH as dilute solvents at different concentrations (a) and etching times (b).*

*Table S5. Sample mass loss of HKUST 72h pH 2.6 repeated 5 times.*

| HKUST 72h pH 2.6 sample | Empty tube (g) | Tube + sample (before) (g) | Sample (before) (g) | Tube + sample (after) (g) | Sample (after) (g) | Sample mass lost (g) | Delta R (g) |
| --- | --- | --- | --- | --- | --- | --- | --- |
| Batch 1 | 14.3037 | 14.5080 | 0.2043 | 14.4576 | 0.1539 | 0.0504 | 0.0020 |
| Batch 2 | 14.2790 | 14.4993 | 0.2203 | 14.4466 | 0.1676 | 0.0527 | 0.0043 |
| Batch 3 | 14.3551 | 14.5715 | 0.2164 | 14.5261 | 0.1710 | 0.0454 | 0.0030 |
| Batch 4 | 14.3073 | 14.5111 | 0.2038 | 14.4654 | 0.1581 | 0.0457 | 0.0027 |
| Batch 5 | 14.3274 | 14.5268 | 0.1994 | 14.4791 | 0.1517 | 0.0477 | 0.0007 |
|  |  |  |  |  |  | ***0.0484*** | ***0.0030*** |

**References**

1. Warden, A. C., Warren, M., Hearn, M. T. W. & Spiccia, L. Binding of inorganic oxoanions to macrocyclic ligands: Effect of the degree of protonation on supramolecular assemblies formed by phosphate and [18]aneN6. *Inorg. Chem.* **43,** 6936–6943 (2004).

2. Koo, J. *et al.* Hollowing out MOFs: Hierarchical micro- and mesoporous MOFs with tailorable porosity via selective acid etching. *Chem. Sci.* **8,** 6799–6803 (2017).

3. Greeves, N. ChemTube3D. Available at: http://www.chemtube3d.com/solidstate/MOF-HKUST-1.html. (Accessed: 23rd January 2019)

4. Zhang, W. *et al.* Impact of Synthesis Parameters on the Formation of Defects in HKUST-1. *Eur. J. Inorg. Chem.* **2017,** 925–931 (2017).

5. Barin, G. *et al.* Defect Creation by Linker Fragmentation in Metal–Organic Frameworks and Its Effects on Gas Uptake Properties. *Inorg. Chem.* **53,** 6914–6919 (2014).

6. Shöâeè, M., Agger, J. R., Anderson, M. W. & Attfield, M. P. Crystal form, defects and growth of the metal organic framework HKUST-1 revealed by atomic force microscopy. *CrystEngComm* **10,** 646 (2008).

7. Kim, S.-Y., Kim, A.-R., Yoon, J. W., Kim, H.-J. & Bae, Y.-S. Creation of mesoporous defects in a microporous metal-organic framework by an acetic acid-fragmented linker co-assembly and its remarkable effects on methane uptake. *Chem. Eng. J.* **335,** 94–100 (2018).

8. Taddei, M. When defects turn into virtues: The curious case of zirconium-based metal-organic frameworks. *Coord. Chem. Rev.* **343,** 1–24 (2017).

9. Park, J., Wang, Z. U., Sun, L.-B., Chen, Y.-P. & Zhou, H.-C. Introduction of Functionalized Mesopores to Metal–Organic Frameworks via Metal–Ligand–Fragment Coassembly. *J. Am. Chem. Soc.* **134,** 20110–20116 (2012).

**List of Figures**

Figure S1. Experimental details of HKUST-1 etching in phosphoric acid using DMSO and MeOH as dilute solvents.

*Figure S2. Experimental details of HKUST-1 etching in phosphoric acid using water as a dilute solvent*

*Figure S3. PXRD and SEM results of HKUST-1 etching in phosphoric acid using water as a dilute solvent (HKUST AEW) in different concentrations (a) and times (b).*

*Figure S4. PXRD results of simulated hydrate and dehydrate HKUST-1 generated by CrystalMaker and CrystalDiffract, normalised to the height of the peak at 11.6 degrees 2*$\theta$*. a) PXRD of both samples in 5-60 degrees 2*$\theta.$*b) Peaks at 11.6 degrees 2*$\theta$*in both patterns are identical. c) Peak at 5.8 degrees 2*$\theta$ *shown in hydrate HKUST-1 has much higher intensity than the dehydrated state.*

*Figure S5. Structure of HKUST-1 visualised using ChemTube3D.*^3^ *The spheres represent the pore sizes within the framework.*

Figure S6. TGA results of HKUST-1 etching in phosphoric acid using DMSO and MeOH as dilute solvents at different concentrations (a) and times (b). Increasing time and decreasing pH are illustrated by the colours from lightest to darkest.

*Figure S7. ^31^P NMR of HKUST-1 etching in phosphoric acid using DMSO and MeOH as dilute solvents at different concentrations (a) and etching times (b).*

**List of Tables**

Table S1. Chemicals used.

*Table S2. BET surface areas of HKUST-1 (measured via N_2_ sorption at 77 K) etching in phosphoric acid using water as a dilute solvent (HKUST AEW) at different concentrations.*

*Table S3. BET surface areas of HKUST-1 (measured via N_2_ sorption at 77 K) etching in phosphoric acid using water as a dilute solvent (HKUST AEW) in different times.*

*Table S4. Gas sorption results of HKUST-1 etching in phosphoric acid using DMSO and MeOH as dilute solvents at different concentrations and times.*

*Table S5. Sample mass loss of HKUST 72h pH 2.6 repeated 5 times.*
